# A multi-index evaluation system for tuff-asphalt mixtures exposed to long-term water damage based on the fractional grey prediction model

**DOI:** 10.1371/journal.pone.0327853

**Published:** 2025-07-10

**Authors:** Yanxia Cai, Jiaming Zhao, Chenchen Li, Jiachen Shi, Baoxin Zhang

**Affiliations:** 1 School of Civil Engineering, Hebei University of Engineering, Handan, Hebei, China; 2 Research Institute of Highway Ministry of Transport, Beijing, China; 3 Zhonglu High Tech (Beijing) Highway Technology Co., Ltd., Beijing, China; 4 Zhejiang Communications Investment Group Expressway Construction and Management Co., Ltd., Hangzhou, Zhejiang, China; 5 School of Traffic and Transportation Engineering, Changsha University of Science & Technology, Changsha, Hunan, China; Shandong University of Technology, CHINA

## Abstract

Given the high susceptibility of tuff-asphalt mixtures to water damage, it is crucial to conduct a comprehensive evaluation of their post-damage performance across multiple performance metrics and integrate these indicators to assess the impact of different water-stability improvement methods on the overall performance of the mixture. This study enhances the long-term water stability of tuff asphalt mixtures by incorporating cement and anti-stripping agents, evaluating their performance under prolonged water exposure. A fractional grey prediction model (FGM) was employed to predict long-term behavior, while a comprehensive evaluation framework was developed using weighted averaging and relative scoring methods. Results demonstrate that both additives significantly improve water stability. Water damage leads to an increase in the viscous components and a decrease in the elastic recovery capacity of asphalt mixtures, which directly results in a decline in their high-temperature rutting resistance. The FGM exhibits high accuracy, with prediction errors below 3% compared to experimental data. The proposed evaluation system provides a practical reference for holistic performance assessment and alternative selection in engineering applications.

## Introduction

Highway construction has developed rapidly in recent years [1]. The consumption of stone materials has increased, and a decrease in the reserves of neutral and alkaline high-quality stone materials has adversely affected highway construction. Abundant reserves of acidic stone materials exist in world [2–4]. They are easily obtained locally and have low costs. The abundant availability and low cost of acidic aggregates can reduce costs in highway construction. Therefore, conducting in-depth research on the application of tuff and other acid aggregates in asphalt pavements holds significant economic and engineering value.

Previous studies have demonstrated poor adhesion between tuff and asphalt [5,6]. Therefore, addressing the water stability of tuff asphalt mixtures has become a top priority. Researchers have found that the addition of silane coupling agents, liquid anti-stripping agents, and cement can effectively enhance the adhesion between tuff and asphalt [7,8], thereby improving the water stability of the asphalt mixture.

The service life of asphalt pavements is a long-term process, yet the duration of water damage tests in laboratories is often relatively short. Consequently, researchers have begun exploring methods to validate the performance of asphalt mixtures under long-term water damage conditions. Zhang et al. [9] evaluated the long-term water stability of asphalt mixtures using splitting tensile strength after seven freeze-thaw cycles. He et al. [10] applied the principle of time-temperature equivalence to simulate long-term water soaking of asphalt mixtures, assessing their long-term water stability through post-soaking splitting tensile and compressive strengths. Huang et al. [11] further employed freeze-thaw-drying cycles (five repetitions) to evaluate the long-term water stability of granite asphalt mixtures via splitting tensile strength. Synthesizing these studies reveals that researchers predominantly utilize long-term water soaking or freeze-thaw cycling to model long-term water damage, and adopt mechanical indices like splitting tensile strength and compressive strength to evaluate water stability. While such mechanical metrics effectively reflect water stability, water damage also significantly impacts other critical properties – including rutting resistance and fatigue resistance. Evaluating water stability solely through mechanical performance therefore represents an overly narrow analytical framework. Therefore, this article not only analyzes the mechanical perspective but also studies the effects of long-term water damage on the high-temperature anti-rutting performance and medium-low temperature anti-cracking performance of tuff asphalt mixture.

The actual service life of asphalt pavements can reach 8–15 years. Considering the temporal limitations of laboratory water damage simulation, this study introduces a grey prediction model to further predict the long-term performance of asphalt mixtures. The grey system theory was proposed by Professor Deng Julong in 1982 [12]. This theory has attracted extensive attention from scholars worldwide due to its unique advantages in the fields of complex system prediction and decision-making [13–16]. The grey prediction model, a component of the grey system theory [17], processes raw data using sequential operators. It reduces the randomness of raw data and detects patterns in the data. It uses discrete raw data to establish dynamic differential equations to predict results. The fractional gray forecast model (FGM) can improve forecast accuracy by handling small sample data [18–21]. The existing studies have demonstrated a significant research gap regarding the applicability of fractional-order grey prediction models in predicting the performance of civil engineering materials. Specifically, for asphalt mixtures-a critical material in road engineering-their long-term properties are subject to pronounced time-dependent characteristics due to multi-factor coupled effects. Traditional prediction methodologies have shown limitations in both accuracy and reliability when applied to such complex scenarios. Therefore, this study intends to construct a fractional-order grey prediction model and verify the applicability of this method in predicting the long-term performance of asphalt mixtures through experimental data, providing new methodological support for the life-cycle management of highway infrastructure.

The research involves diverse and mutually independent performance metrics, yet holistic integration of all performance aspects remains challenging in practical engineering applications. Different projects typically prioritize distinct performance criteria, which hinders comprehensive evaluation and comparison of alternative schemes. Currently, scholars have conducted limited research on the integration of multiple performance metrics. This study investigates the feasibility of combining different evaluation indicators and establishes a long-term water damage evaluation system for asphalt mixtures using weighted averages. Given the elevated temperatures characteristic of humid and hot regions, asphalt pavements maintain relatively high temperatures even during winter [22,23]. Considering the inclusion of long-term water damage factors in the experimental design, this method prioritises the weighting of asphalt mixture performance at high and medium temperatures, significantly reduces the allocation of weight to low-temperature performance, and moderately adjusts the weight assigned to the mechanical splitting test. This method is aimed at providing theoretical and technical support for the long-term durability of asphalt pavements in humid-thermal regions.

In summary, current research faces two main issues: short duration of water damage simulation and limited evaluation metrics. This study proposes to address these issues by conducting laboratory tests to simulate water damage and combining the results with FGM predictions. Additionally, a new comprehensive evaluation framework is established to overcome the challenges in comprehensive performance evaluation and scheme comparison.

## Materials and methods

### Raw material

The test results reveal that the uniaxial compressive strength (UCS) of tuff is 118.22 MPa, indicating relatively high mechanical integrity. The chemical composition of the tuff aggregate was analyzed using an X-ray diffractometer. Flocculent lignin fiber was used as an additive. Its content (0.3%) was selected based on engineering experience. An anti-stripping agent (phosphate ester) and P·O 42.5 ordinary Portland cement were used. The technical properties of the raw materials were tested according to Chinese standards [24,25] (Tables 1–5).

**Table 1:** The technical properties of SBS-modified asphalt.

Parameter	Test result
**Penetration (25°C;0.1 mm)**	53
**Ductility(5°C;cm)**	35
**Softening point(°C)**	90.5
**Dynamic viscosity(135°C,Pa·s)**	2.5

**Table 2:** The technical properties of coarse aggregate.

Parameter	Tuff		Basalt	
	9.5-13.2 mm	4.75-9.5 mm	9.5-13.2 mm	4.75-9.5 mm
**Apparent specific gravity**	2.691	2.690	2.923	2.920
**Flakiness and elongation (%)**	6.3	6.4	5.3	4.0
**Water absorption (%)**	0.49	0.66	0.71	1.33
**Los Angeles abrasion (%)**	11.4		13.8	
**Crush value (%)**	9.2		11.8	

**Table 3:** The technical properties of fine aggregate.

Parameter	Limestone
**Apparent specific gravity**	2.736
**Sand equivalent (%)**	72
**Methylene blue value(g/kg)**	1.0
**Fine aggregate angularity(s)**	31.0

**Table 4:** The chemical composition of the aggregate.

Sample	Average content of main components(%)						
	SiO_2_	Al_2_O_3_	Na_2_O	Fe_2_O_3_	K_2_O	CaO	MgO
**Tuff**	66.81	16.62	4.63	3.34	3.29	3.17	1.32
**Basalt**	47.70	14.45	2.66	12.33	0.99	7.87	8.28
**Limestone**	13.60	1.48	0.35	0.55	0.24	49.68	33.80

**Table 5:** The chemical composition of limestone powder.

Parameter	Test result
**Apparent density(g/cm3)**	2.736
**Hydrophilicity coefficient**	0.7
**Water content (%)**	0.5

### Mix proportions

The mix proportions of the SMA-13 asphalt mixture, the mineral aggregate gradation, and the optimal asphalt aggregate ratio according to Chinese standards [24] are listed in Table 6.

**Table 6:** Target gradation and optimal asphalt aggregate ratio of the asphalt mixture.

/	Passing rate (%)										Oil stone ratio (%)
**Sieve aperture**	16	13.2	9.5	4.75	2.36	1.18	0.6	0.3	0.15	0.075	/
**Basalt**	100	97.6	59.9	24.6	19.6	15.1	13.3	12.0	11.5	9.7	6.0
**Tuff**	100	98.3	59.6	28.9	20.8	16.3	14.4	13.1	12.6	10.6	6.2

### Experimental and analytical methods

**Fig 1:**
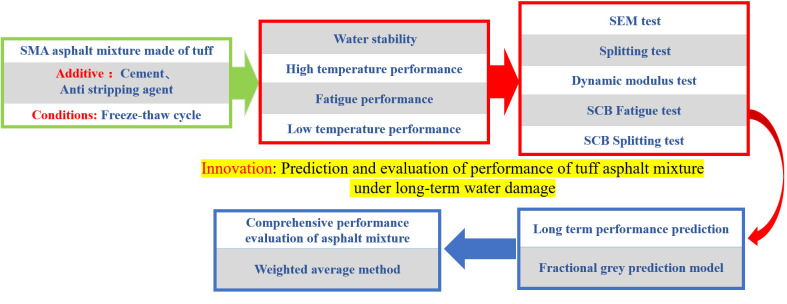
The research framework.

We used multiple freeze-thaw cycles to simulate the long-term water damage to asphalt mixtures. The high-, medium-, and low-temperature performances of the asphalt mixture with water damage were evaluated using the anti-rutting factor ($|E^{*}|/sin\varphi$), fatigue life, and fracture energy, respectively. The freeze-thaw splitting tensile strength ratio (TSR) was used to evaluate the resistance to water damage of the asphalt mixture. The FGM was used to predict the performance of the asphalt mixture after long-term water damage, and the weighted average method was applied to compare the performance of different asphalt mixtures. According to results obtained from previous experiments conducted by our research group, the dosage of the anti-stripping agent was 0.6% of the asphalt mass, and the limestone powder was replaced with 20% of the mass of the cement. The research framework is as shown in Fig 1, and the experimental plan is presented in Table 7.

**Table 7:** Asphalt mixture test protocol.

Protocol Code	Coarse aggregate	Fine aggregate	Filler	Asphalt
**B**	Basalt	Limestone	Limestone powder	SBS modified asphalt
**T**	Tuff	Limestone	Limestone powder	SBS modified asphalt
**TC**	Tuff	Limestone	Limestone powder (20% cement)	SBS modified asphalt
**TA**	Tuff	Limestone	Limestone powder	SBS modified asphalt (0.6% Anti stripping agent)

The specimens were fully saturated with water under vacuum conditions of 97.3–98.7 kPa and frozen at −18 °C for 24 hours. Subsequently, they were immersed in a constant-temperature water bath at 60 °C for 24 hours and subjected to 0, 1, 3, 5, and 7 freeze-thaw cycles. The specimens were vacuum-saturated before being placed in the freezer each time to ensure that no moisture was lost during the freeze-thaw cycle.

#### Resistance to water damage.

The Marshall specimens were compacted 50 times. After the freeze-thaw cycles, the specimens were subjected to splitting tests at a loading rate of 50 mm/min to calculate the TSR and evaluate the long-term resistance of the mixtures to water damage. After the splitting test, we obtained aggregates adhering to the asphalt from the interior of the damaged specimen for scanning electron microscopy (SEM) analysis.

#### High-temperature performance.

**Fig 2:**
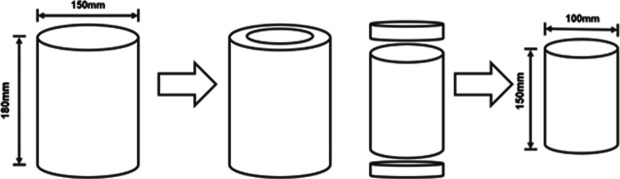
Fabrication of cylindrical specimens.

A cylindrical specimen with a diameter of 150 mm and a height of 180 mm was fabricated using a rotary compactor. A core sample with a diameter of 100 mm was obtained using a core drilling machine, and the parts with large void ratios at both ends of the specimen were removed using a cutting machine to ensure a height of 150 mm. The process is shown in Fig 2.

After the freeze-thaw cycles, the specimens were cured at 60 °C for 5 hours. They were transferred to a Simple Performance Test (SPT) instrument and kept at 60 °C for 0.5 hours before undergoing dynamic uniaxial compression testing. The dynamic modulus ($\left|E^{*}\right|$) and phase angle (φ) of the specimens were obtained at loading frequencies of 25 Hz, 20 Hz, 10 Hz, 5 Hz, and 1 Hz, and the anti-rutting factor $|E^{*}|/sin\varphi$ was calculated [26,27].

#### Fatigue performance.

**Fig 3:**
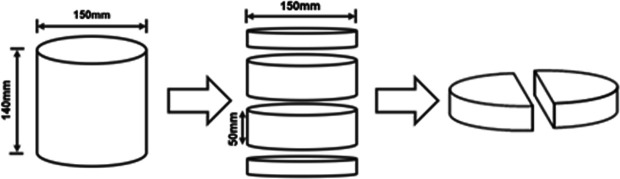
Fabrication of semi-circular specimens.

A cylindrical specimen with a diameter of 150 mm and a height of 140 mm was fabricated using a rotary compactor. After removing the parts with large void ratios at both ends of the specimen using a cutting machine, the remaining parts were cut into four semi-circular specimens with a thickness of 50 mm. The process is shown in Fig 3.

After the freeze-thaw cycles, the specimens were cured at 15 °C for 5 hours and were transferred to a multifunctional material testing machine equipped with a temperature control box for the asphalt mixture. The stress ratio and loading frequency were 0.3 and 10 Hz, respectively. The specimens were loaded using a sine wave until failure, and the number of loading cycles represented the fatigue life of the asphalt mixture [28,29].

#### Low-temperature performance.

We used the same method as above to create a semi-circular specimen. After the freeze-thaw cycle, the specimens were cured at −10 °C for 5 hours and transferred into a multifunctional asphalt mixture testing machine equipped with a temperature control box and subjected to a splitting test at a loading rate of 50 mm/min. The low-temperature fracture energy of the asphalt mixture was calculated using Eq. (1) [30,31].


$$\begin{array}{c} G_{f}=W_{f}/A_{lig} \end{array}$$ (1)

where $G_{f}$ is the fracture energy, J/m^2^; $W_{f}$ is the fracture energy, J; $A_{lig}=r\times t$ is the fracture surface area, m^2^; r is the radius of the sample, m; t is the thickness of the sample, m.

### Fractional grey prediction model

We predicted the properties of the asphalt mixtures after long-term water damage using the FGM (1,1). This uses a small sample size to establish a prediction model. The most common model is the GM (1,1). Due to the low accuracy of the GM (1,1) model, researchers have developed the FGM and selected appropriate fractional orders to improve the prediction accuracy. The FGM (1,1) uses a fractional order accumulation operator to reduce the randomness of the data sequence and the effects on the solution and improve the fitting accuracy. The basic principle of the FGM (1,1) is as follows [32–34]:

The original sequence $X^{\left(0\right)}=\{x^{\left(0\right)}(1),x^{\left(0\right)}(2){,\cdots ,x}^{\left(0\right)}(n\}$, and the accumulation equation $x^{\left(r\right)}(k)=\sum_{i=1}^{k}C_{k-i+r-1}^{k-i}x^{\left(0\right)}(i)$, $C_{r-1}^{0}=1$,$C_{k}^{k+1}=0$,$C_{k-i+r-1}^{k-i}=(k-i+r-1)(k-i+r-2cdots(r+1)r/(k-i)!$ is used to obtain the *r* th-order accumulation sequence $X^{\left(r\right)}$ for $X^{\left(0\right)}$:


$$\begin{array}{c} X^{\left(r\right)}=\left\{x^{\left(r\right)}(1),x^{\left(r\right)}(2){,\cdots ,x}^{\left(r\right)}(n)\right\} \end{array}$$ (2)

The sequence to generate the mean $Z^{\left(r\right)}$ of $X^{\left(r\right)}$ is


$$\begin{array}{c} Z^{\left(r\right)}=\left\{z^{\left(r\right)}(2),z^{\left(r\right)}(3){,\cdots ,z}^{\left(r\right)}(n)\right\} \end{array}$$ (3)

The background value $z^{\left(r\right)}(k)$ is


$$\begin{array}{c} z^{\left(r\right)}(k)=0.5\left\{x^{\left(r\right)}(k)+x^{\left(r\right)}(k-1)\right\},k=2,3,\cdots ,n \end{array}$$ (4)

The basic form of the FGM (1,1) is called b


$$\begin{array}{c} x^{\left(r\right)}(k)-x^{\left(r\right)}(k-1)+az^{\left(r\right)}(k)=b \end{array}$$ (5)

The whitening equation of the FGM (1,1) is Eq. (6)


$$\begin{array}{c} {dx}^{\left(r\right)}(t)/dt+ax^{\left(r\right)}(t)=b \end{array}$$ (6)

where a is the development coefficient, and b is the grey action quantity.

If the parameter vector $\hat{\beta }={\left[\hat{a},\hat{b}\right]}^{T}$,B  and Y are defined as follows:


$$\begin{array}{c} B=[\begin{array}{c} -0.5\left(x^{\left(r\right)}(1)+x^{\left(r\right)}(2)\right)\\ -0.5\left(x^{\left(r\right)}(2)+x^{\left(r\right)}(3)\right)\\ \vdots \\ -0.5\left(x^{\left(r\right)}(n-1)+x^{\left(r\right)}(n)\right) \end{array}\;\;\begin{array}{c} 1\\ 1\\ \vdots \\ 1 \end{array}],Y=\left[\begin{array}{c} x^{\left(r\right)}(2)-x^{\left(r\right)}(1)\\ x^{\left(r\right)}(3)-x^{\left(r\right)}(2)\\ \vdots \\ x^{\left(r\right)}(n)-x^{\left(r\right)}(n-1) \end{array}\right] \end{array}$$ (7)

The least squares estimation parameter of the grey differential equation $x^{\left(0\right)}(k)+az^{\left(r\right)}(k)=b$ satisfies


$$\begin{array}{c} \hat{\beta }={\left(B^{T}B\right)}^{-1}B^{T}Y \end{array}$$ (8)

Under the initial condition $x^{\left(0\right)}(1)=x^{\left(r\right)}(1)$, the solution of the whitening equation${dx}^{\left(r\right)}(t)/dt+ax^{\left(r\right)}(t)=b$ (also known as the time response function) is


$$\begin{array}{c} x^{\left(r\right)}(t)=\left[x^{\left(0\right)}(1)-\hat{b}/\hat{a}\right]\exp[-\hat{a}(t-1)]+\hat{b}/\hat{a} \end{array}$$ (9)

The time response sequence of the grey differential equation $x^{\left(0\right)}(k)=+az^{\left(r\right)}(k)=b$of FGM (1,1) is


$$\begin{array}{c} {\hat{x}}^{\left(r\right)}(k)=\left[x^{\left(0\right)}(1)-\hat{b}/\hat{a}\right]\exp[-\hat{a}(t-1)]+\hat{b}/\hat{a},k=2,3,\cdots ,n \end{array}$$ (10)

where ${\hat{x}}^{\left(r\right)}(k)$ is the value at time k. A reduction operator is used for ${\hat{x}}^{\left(r\right)}(k)$ to obtain the following expression


$$\begin{array}{c} {\hat{x}}^{0}(k)=\alpha^{\left(r\right)}{\hat{x}}^{\left(r\right)}(k)={\hat{x}}^{\left(r)(1-r\right)}(k)-{\hat{x}}^{\left(r)(1-r\right)}(k-1) \end{array}$$ (11)

### Particle swarm optimization

Different cumulative orders produce different prediction accuracies of FGMs; thus, finding the optimal order is crucial. However, traditional optimization methods often cannot determine the optimal order. Therefore, researchers have used the particle swarm optimization algorithm.

The model steps of the particle swarm optimization algorithm are as follows [35–37]. It is assumed that m particles exist in the D-dimensional solution space. Each particle is a potential solution to the optimization function. The velocity and position of the i-th particle are denoted as $V_{i}=(v_{i}^{1},v_{i}^{2}{,\cdots ,v}_{i}^{D})$ and $X_{i}=(x_{i}^{1},x_{i}^{2}{,\cdots ,x}_{i}^{D})$, respectively. The fitness of the particle is obtained by substituting $X_{i}$ into the optimization function. During the iteration, the individual extremum is denoted as $P_{i}=(p_{i}^{1},p_{i}^{2}{,\cdots ,p}_{i}^{D})$, and the optimal position of the particle is denoted as $P_{g}=(p_{g}^{1},p_{g}^{2}{,\cdots ,p}_{g}^{D})$. The velocity and position of the particles are updated during the iterations. The updated equations are Eqs. (12) and (13).


$$\begin{array}{c} V_{i+1}=wv_{i}^{d}=c_{1}r_{1}\left(p_{i}^{d}-x_{i}^{d}\right)+c_{2}r_{2}\left(p_{g}^{d}-x_{i}^{d}\right) \end{array}$$ (12)

$$\begin{array}{c} x_{i+1}=x_{i}+v_{i} \end{array}$$ (13)

where $V_{i+1}$ represents the velocity of the particles after the iterative updates; wis the inertia factor; $r_{1}$ and $r_{2}$ are random numbers from 0 to 1; $c_{1}$ and $c_{2}$ are learning factors; $x_{i+1}$ is the position of the particle after the iterative update.

The conditions for terminating the iteration depend on the optimization function. Generally, the number of iterations to determine the optimal position meets the minimum threshold or the maximum number of iterations is used.

## Analysis of long-term water damage

### Evaluation of long-term resistance to water damage

**Fig 4:**
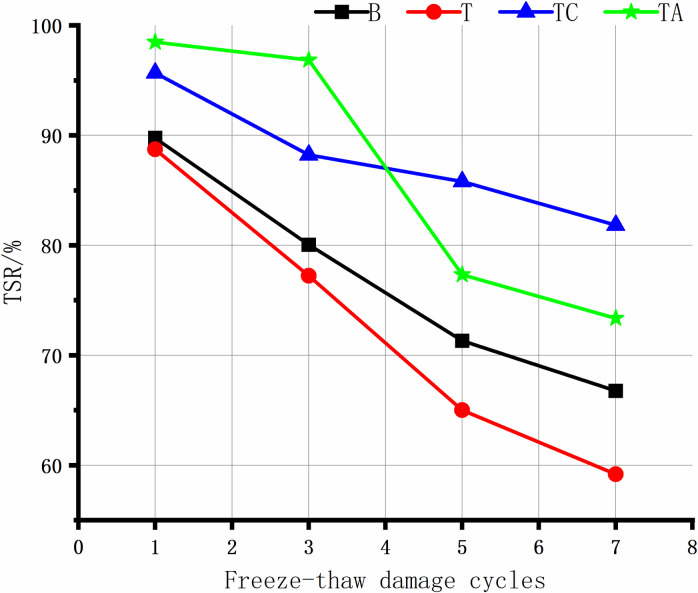
TSR of asphalt mixtures after freeze-thaw cycles.

The adhesion between acidic tuff and asphalt is low, and the material is prone to water damage. Therefore, assessing the long-term resistance of the tuff-asphalt mixture to water damage is crucial. The TSR of the asphalt mixtures after the freeze-thaw cycles is shown in Fig 4.

As the number of freeze-thaw cycles increases, the TSR of the four asphalt mixtures shows a decreasing trend. T exhibits the largest freeze-thaw cycle damage, followed by B. The cement and anti-stripping agents significantly increase the tuff-asphalt mixtures’ water damage resistance.

The anti-stripping agents improve the water damage resistance of the mixture more significantly in the early stage of water damage. After the exposure to three freeze-thaw cycles, the TSR of TA is 19.62% and 8.64% higher for TA than for T and TC, respectively. However, the TSR of TA is much lower after five freeze-thaw cycles. The reason is that the anti-stripping agent improves the adhesion between the asphalt/aggregate interface, and bonding products are generated between the modified surface of the tuff aggregate and the asphalt, resulting in higher early resistance to water damage. As the number of freeze-thaw cycles increases, the asphalt becomes more susceptible to damage due to the anti-stripping agents. The interfacial bonding products break down, rapidly decreasing the mixture’s strength in the later stage.

The strength of TC decreases the slowest during the freeze-thaw cycles. After 5 freeze-thaw cycles, the TSR of TC is higher (85.80%) than that of TA. The cement undergoes a hydration reaction, delaying the occurrence of water damage, and the hydration products themselves improve the mixture’s strength due to their skeleton, resulting in a slow decay in the TSR strength of the TC mixture.

### Analysis of water damage mechanism

**Fig 5:**
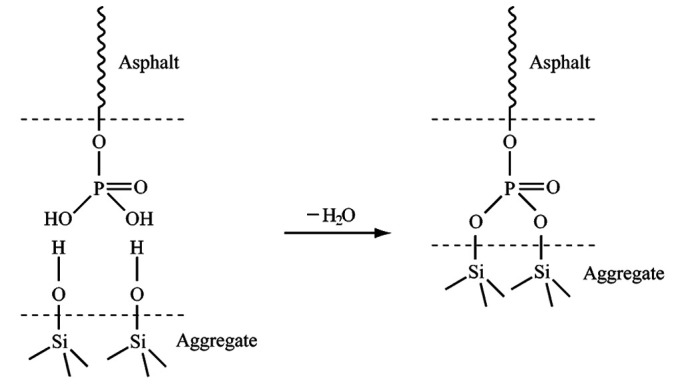
Schematic diagram of the overlapping effect of the anti-stripping agent.

As shown in Fig 5, one end of the phosphate ester in the anti-stripping agent is a polar phosphate hydroxyl group, and the other end is an alkyl group. Phosphate hydroxyl groups undergo dehydration reactions with the hydroxyl groups in the tuff aggregates, causing them to be adsorbed uniformly on the aggregate surface. The alkyl groups attract the oil molecules in the asphalt. The anti-stripping agents contain phosphate surfactants, forming bridges at the oil-stone interface, improving the adhesion between the asphalt and tuff surface and resulting in a strong bond [38].

The cement reacts with carboxylic acids and p-diphenol in asphalt, generating new functional groups and reducing the polarity of asphalt and improving the adhesion between asphalt and aggregates. The following reactions occur during the water damage process [39]:


$$\begin{array}{c} {Ca(OH)}_{2}+{CO}_{2}\to {CaCO}_{3}+H_{2}O \end{array}$$ (14)

$$\begin{array}{c} 3CaO\cdot 2{SiO}_{2}\cdot 3H_{2}O+3{CO}_{2}\to 3{CaCO}_{3}+2{SiO}_{2}\cdot 3H_{2}O \end{array}$$ (15)

$$\begin{array}{c} \left[{Ca}_{3}{Al(OH)}_{6}\right]2\left({SO}_{4}\right)3\cdot 26H_{2}O\to 3{CaCO}_{3}+3\left({CaSO}_{4}\cdot 2H_{2}O\right)\cdot {Al}_{2}O_{3}\cdot xH_{2}O\\ \;+(26-x)H_{2}O \end{array}$$ (16)

**Fig 6:**
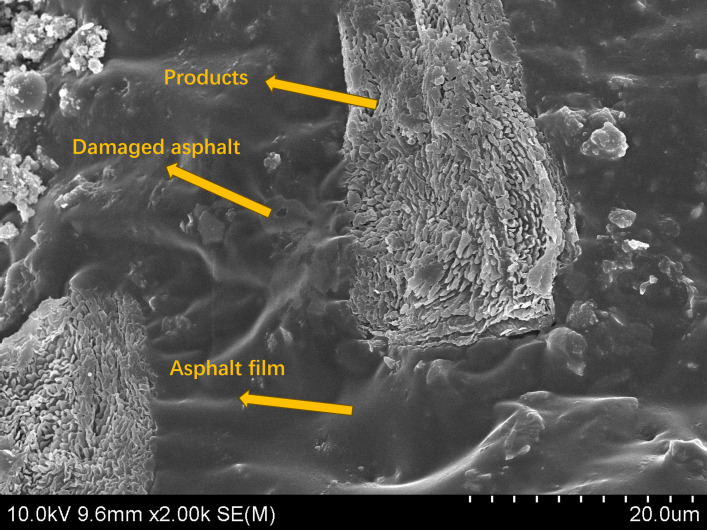
SEM images of the adhesion interface of the asphalt mixture after 7 freeze-thaw cycles of TA.

**Fig 7:**
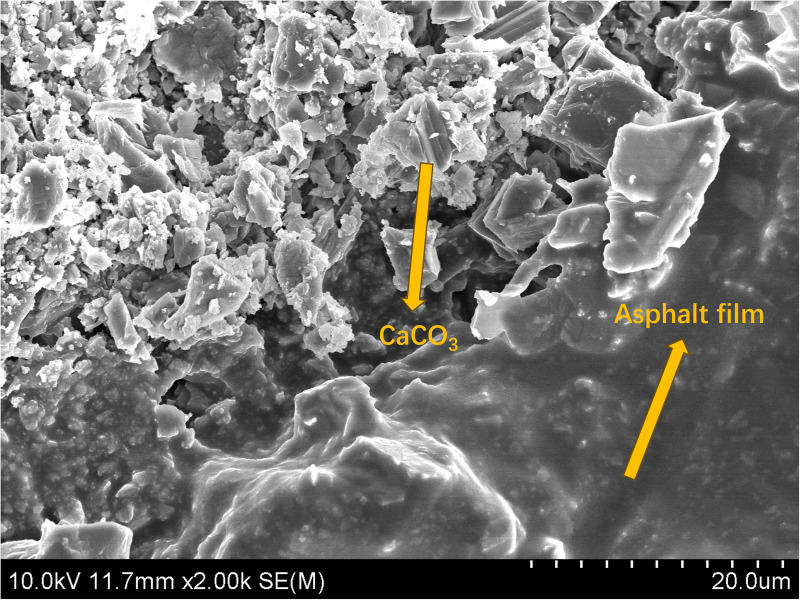
SEM images of the adhesion interface of the asphalt mixture after 7 freeze-thaw cycles of TC.

SEM was used to analyze the Marshall specimens after 7 freeze-thaw cycles and investigate the effect of the two additives on the microstructure and water damage resistance of the asphalt mixture. Damage was observed at the adhesion interface between the asphalt and the aggregate (Figs 6 and 7).

Fig 6 shows that the phosphate esters modified the interface between the asphalt and the tuff, generating many bonding products. The bond between the modified product and the asphalt was damaged after 7 freeze-thaw cycles, and the surrounding asphalt was damaged. A significant color difference and boundary exists between the damaged and undamaged areas of the asphalt, decreasing the mixture’s water damage resistance. Fig 7 shows that a large number of crystalline products were generated between the asphalt and the aggregate. It was determined that the product was CaCO_3_ resulting from cement hydration [39].

## Analysis of road performance after water damage

### High-temperature performance

**Fig 8:**
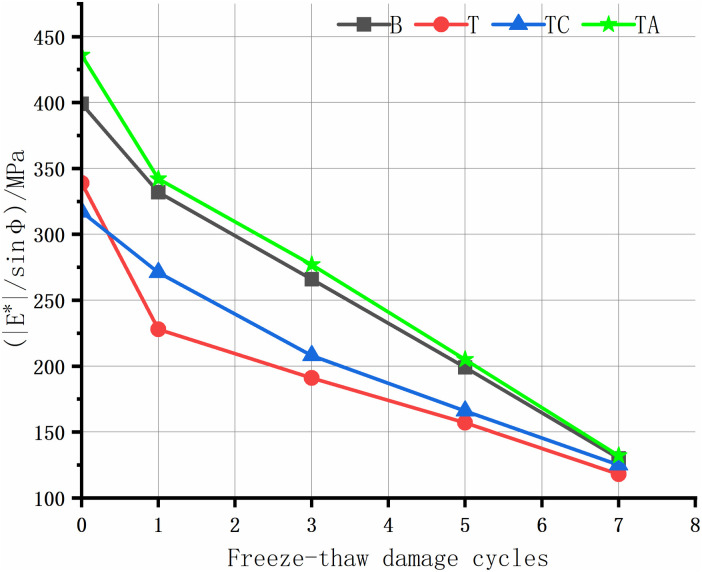
Anti-rutting factors of the asphalt mixture after freeze-thaw cycles(1 Hz).

**Fig 9:**
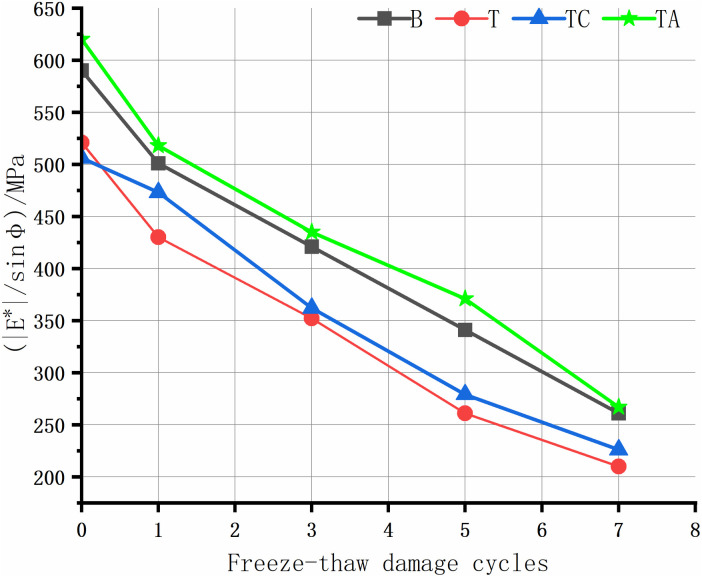
Anti-rutting factors of the asphalt mixture after freeze-thaw cycles(5 Hz).

**Fig 10:**
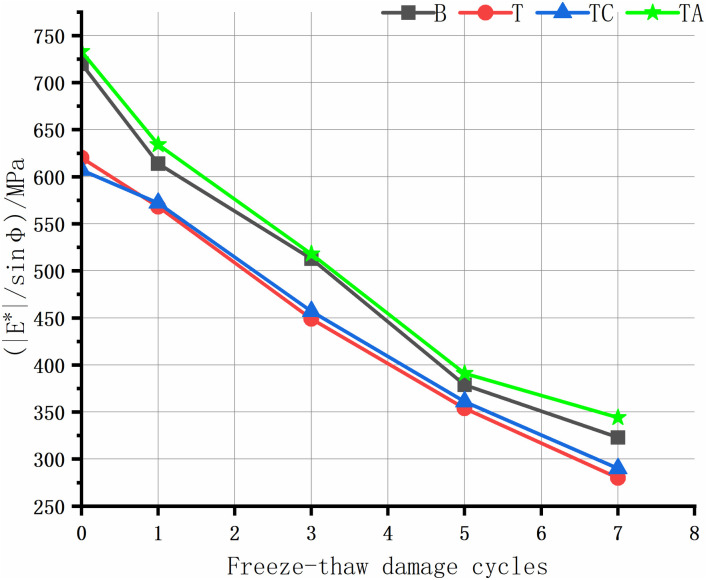
Anti-rutting factors of the asphalt mixture after freeze-thaw cycles(10 Hz).

**Fig 11:**
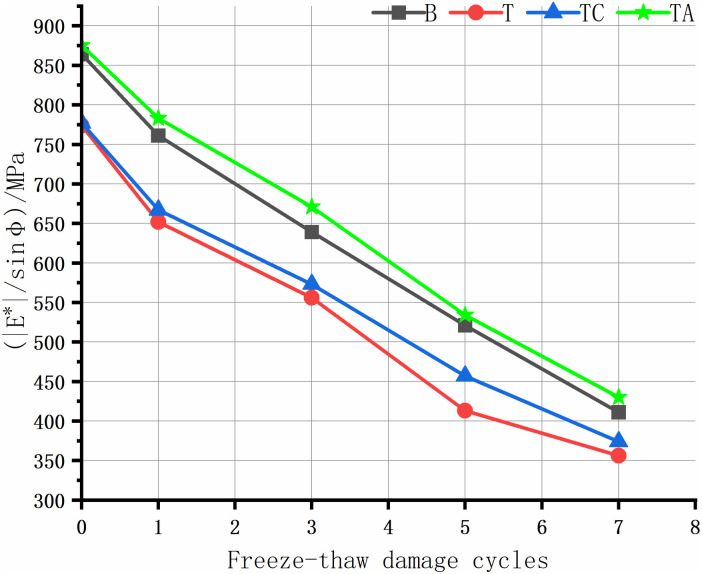
Anti-rutting factors of the asphalt mixture after freeze-thaw cycles(20 Hz).

**Fig 12:**
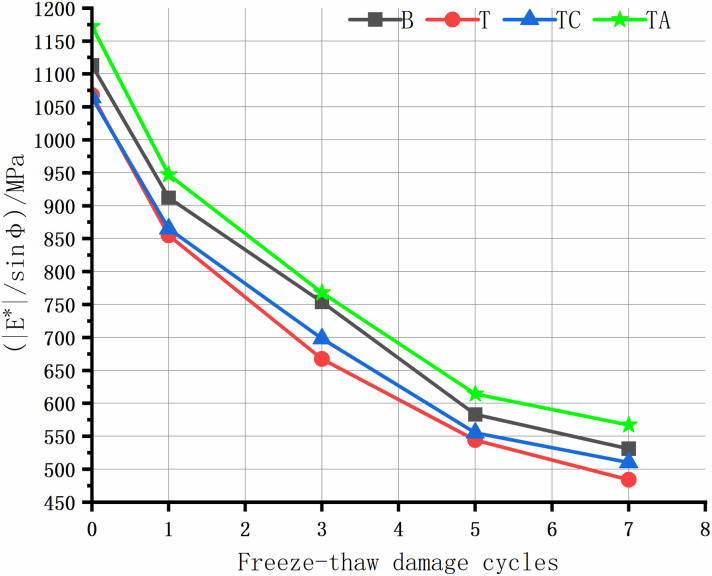
Anti-rutting factors of the asphalt mixture after freeze-thaw cycles(25 Hz).

The rutting of asphalt pavement is the most significant structural pavement damage, especially in areas with high summer temperatures. Asphalt rutting occurs faster under heavy loads; thus, assessing the high-temperature rutting resistance of the asphalt mixture is crucial. The high-temperature anti-rutting factors of the asphalt mixtures after the freeze-thaw cycles are shown in Figs 8–12, and the phase angles of the asphalt mixture after the freeze-thaw cycles at 25 Hz are listed in Table 8.

**Table 8:** Phase angle of the asphalt mixture after the freeze-thaw cycles at 25 Hz.

Test Scheme	Phase angle after different freeze-thaw cycles φ(°)				
	0	1	3	5	7
**B**	28.36	32.44	32.68	34.78	35.37
**T**	29.48	31.32	33.3	35.69	36.23
**TC**	29.7	32.62	34.09	34.35	35.54
**TA**	27.82	32.5	32.9	33.67	34.78

As the level of water damage increases, the anti-rutting factors of the four asphalt mixtures at different frequencies decrease. The anti-stripping agent significantly improves the high-temperature performance of the tuff-asphalt mixture. The anti-rutting factor is higher for the tuff-asphalt mixture than for the basalt/asphalt mixture at all water damage levels. The anti-stripping agent improves the softening of asphalt [40], and the bonding products generated by the agent at the asphalt/aggregate interface cause significant viscous resistance of the mixture, increasing the rutting resistance of the mixture. As the frequency decreases from 25 Hz to 1 Hz, the anti-rutting factor of the mixture not damaged by water improves from 9% to 29.5%. The other asphalt mixtures show a similar trend. As the frequency decreases, the difference in the anti-rutting factors between the different mixtures increases. This finding explains why ruts are more common on ramps, near toll stations, and on municipal roads than highways. The higher the vehicle speed, the less time the wheels remain on the road surface, leaving more time for the elastic recovery of the mixture and resulting in a higher elastic modulus. When the TC is not damaged by water, the cement has almost no effect on the high-temperature performance of the tuff-asphalt mixture. As the number of freeze-thaw cycles increases, the effect of the cement on the water damage resistance of the tuff-asphalt mixture becomes more pronounced. The CaCO_3_ formed by cement hydration increases the mixture’s strength, significantly improving the modulus and anti-rutting factor of the mixture.

The phase angle φ represents the degree to which the response strain of viscoelastic materials lags behind the stress, reflecting the ratio of elastic and viscous components in the material properties. The phase angle of elastic materials is 0°, whereas that of viscous materials is 90° [26]. In this study, the phase angle of the asphalt mixtures in each scheme increases with the number of freeze-thaw cycles. The elasticity is lower after the mixture has been damaged by water. Thus, the asphalt mixture has lower resistance to elastic deformation and rutting. Therefore, the phase angle is negatively proportional to the asphalt mixture’s elasticity and rutting resistance. Due to the same trend in the phase angle at different frequencies, we only describe the results for 25 Hz.

### Fatigue performance

**Fig 13:**
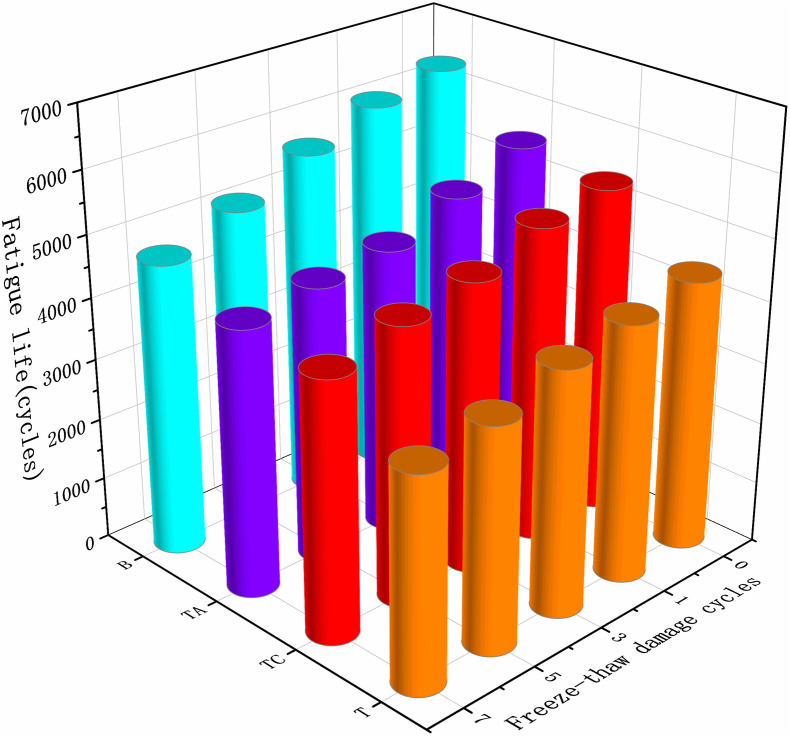
Fatigue life of asphalt mixtures after freeze-thaw cycles.

The asphalt mixture performance deteriorates over time due to repeated vehicle loads and environmental factors, resulting in cumulative damage, cracks, and failure, i.e., fatigue damage. The fatigue life of the asphalt mixtures after the freeze-thaw cycles for different schemes is shown in Fig 13.

The basalt/asphalt mixture has the highest fatigue life, and TA has the highest fatigue life for the tuff-asphalt mixtures, followed by TC and T. The reason is that the relative density is higher for the basalt coarse aggregate (2.923) than for the tuff (2.691). The proportion of lignin fibers added to the asphalt mixture was 0.3%, resulting in fewer lignin fibers in the tuff-asphalt mixture than in the basalt/asphalt mixture for the same volume. Lignin fibers delay the failure of the asphalt mixtures, resulting in higher fatigue life of the basalt/asphalt mixtures than of the tuff-asphalt mixtures. The addition of cement and the anti-stripping agent to the tuff-asphalt mixture makes the asphalt slurry more viscous and increases its viscoelasticity [40], resulting in a stronger ability to recover fatigue deformation and resist fatigue damage.

### Low-temperature performance

**Fig 14:**
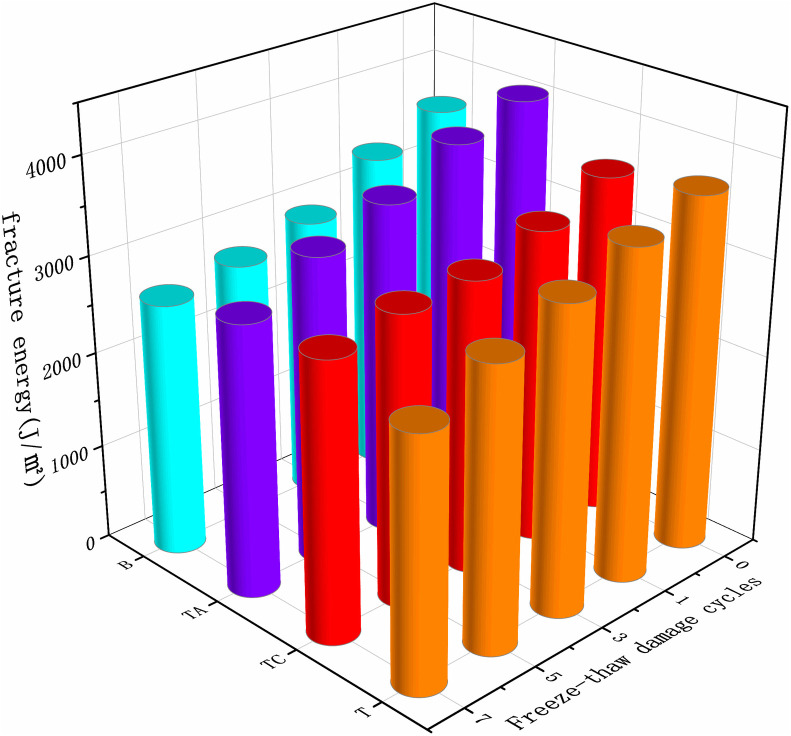
Low-temperature fracture energy of asphalt mixtures after freeze-thaw cycles.

Asphalt pavement cracks under temperature stress in areas with sudden temperature drops or large temperature differences, reducing the service life and road quality. The low-temperature fracture energy of the asphalt mixtures after the freeze-thaw cycles for different schemes is shown in Fig 14.

The low-temperature fracture energy of various asphalt mixtures decreases with the degree of freeze-thaw damage. The difference between the low-temperature fracture energies of T and B is small, indicating that the acidity and alkalinity of the coarse aggregates in the SMA asphalt mixtures have a negligible effect on the low-temperature crack resistance of the asphalt mixtures. The anti-stripping agents improve the low-temperature performance of the asphalt mixtures in the early stages of water damage. The low-temperature performance of TA decreases rapidly by 29.9% during the water damage process. The reason is that the anti-stripping agents significantly improve the low-temperature ductility of asphalt [40]. However, as the number of freeze-thaw cycles increases, the asphalt suffers greater damage, resulting in a significant decrease in the low-temperature performance of the asphalt mixtures. The low-temperature crack resistance of TC deteriorates the slowest due to the formation of CaCO_3_, which increases the strength of the mixture.

## Predicting the long-term performance of the mixture using the fractional grey prediction model

**Fig 15:**
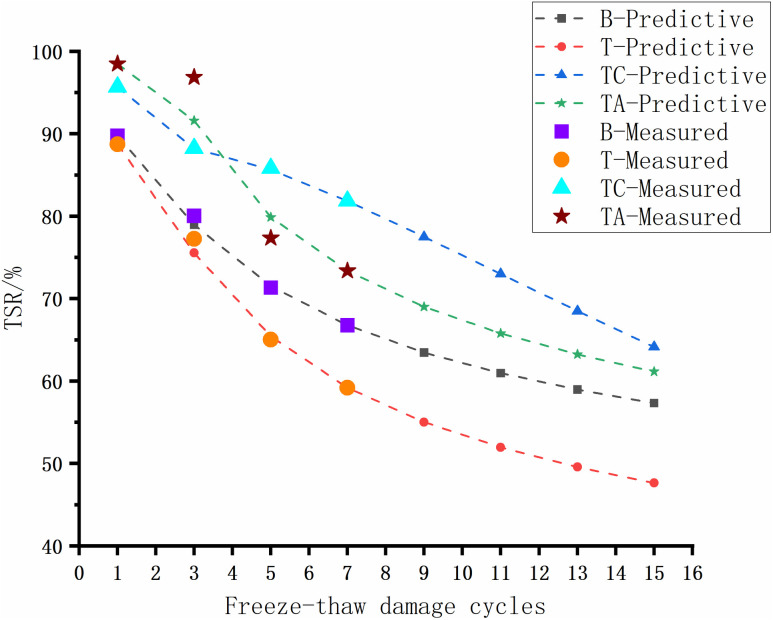
Predicted and experimental results (Water stability).

**Fig 16:**
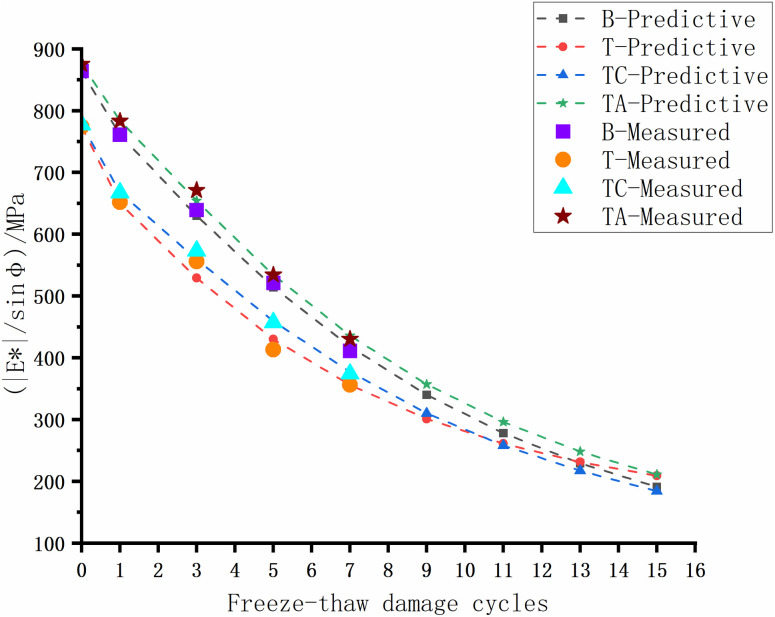
Predicted and experimental results (High temperature performance of 20 Hz).

**Fig 17:**
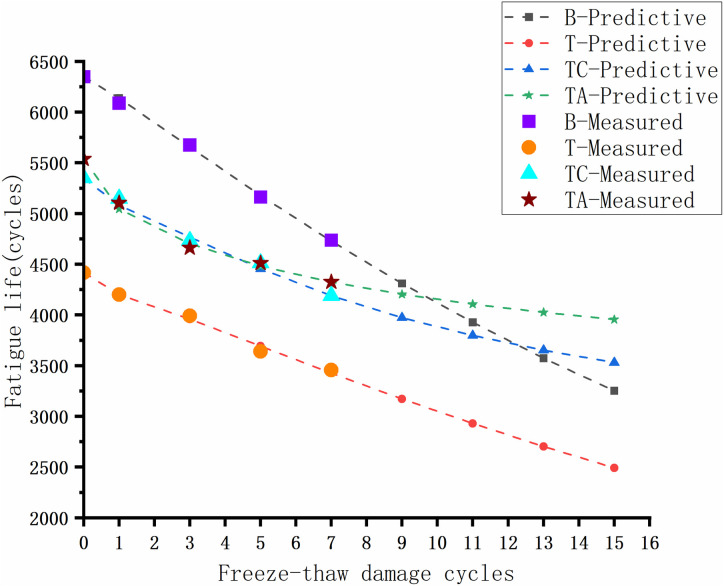
Predicted and experimental results (Fatigue performance).

**Fig 18:**
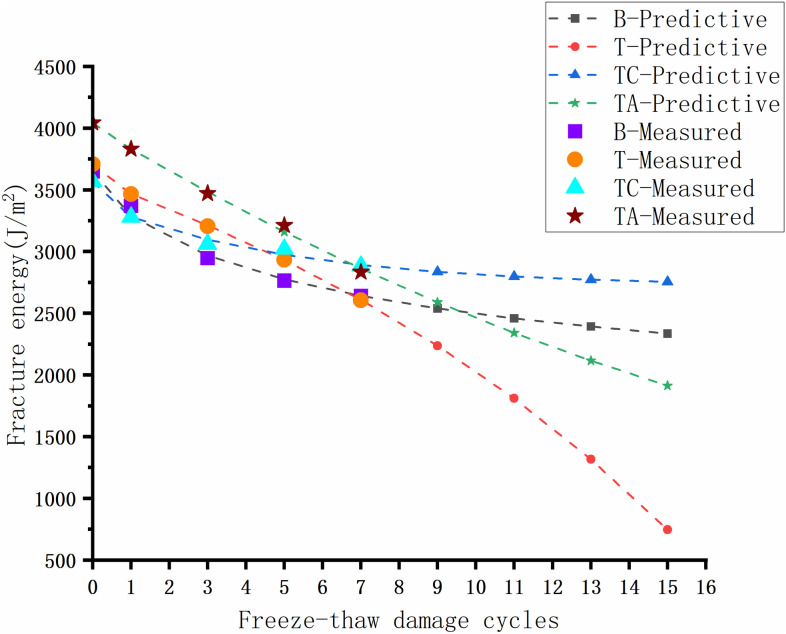
Predicted and experimental results (Low temperature performance).

After obtaining the results of the performance tests, we used the particle swarm optimization algorithm and FGM in MATLAB software to calculate the optimal order and predict the performance of the asphalt mixture after 0–15 freeze-thaw cycles. A driving speed of 120 km/h at 20 Hz reflects driving conditions on highways. Therefore, we used the anti-rutting factor at 20 Hz to perform simulations and predict the high-temperature performance of the asphalt mixtures. After 2000 iterations, the relative error between experimental data and simulation results remains small, with an overall magnitude of less than 3%, achieving a higher accuracy than conventional grey prediction models [41–43]. The predicted and experimental results are shown in Figs 15–18. The results of the prediction model and the optimal fractional order are listed in Tables 9–12.

**Table 9:** Prediction model for resistance to water damage and optimal fractional order.

Protocol Code	Optimal fractional order	Prediction model
**B**	0.191967	${\hat{x}}^{0}(k)=97.0337e^{-2.1345(k-1)}+97.0337$
**T**	0.266976	${\hat{x}}^{0}(k)=100.7977e^{-2.0404(k-1)}+100.7977$
**TC**	0.860495	${\hat{x}}^{0}(k)=866.9039e^{-0.1021(k-1)}+866.9039$
**TA**	0.225782	${\hat{x}}^{0}(k)=114.1753e^{-3.7761(k-1)}+114.1753$

**Table 10:** Prediction model for high-temperature performance and optimal fractional order.

Protocol Code	Optimal fractional order	Prediction model
**B**	0.860423	${\hat{x}}^{0}(k)=3304.9005e^{-0.30431(k-1)}+3304.9005$
**T**	0.608841	${\hat{x}}^{0}(k)=1508.309e^{-0.6405(k-1)}+1508.309$
**TC**	0.788388	${\hat{x}}^{0}(k)=2456.4086e^{-0.35627(k-1)}+2456.4086$
**TA**	0.783558	${\hat{x}}^{0}(k)=2786.7113e^{-0.37259(k-1)}+2786.7113$

**Table 11:** Prediction model for fatigue life and optimal fractional order.

Protocol Code	Optimal fractional order	Prediction model
**B**	0.964087	${\hat{x}}^{0}(k)=65983.4146e^{-0.10435(k-1)}+65983.4146$
**T**	0.932531	${\hat{x}}^{0}(k)=45941.98849e^{-0.09868(k-1)}+45941.9884$
**TC**	0.245713	${\hat{x}}^{0}(k)=7147.0586e^{-0.87399(k-1)}+7147.0586$
**TA**	0.13739	${\hat{x}}^{0}(k)=5829.6251e^{-2.5177(k-1)}+5829.6251$

**Table 12:** Prediction model for low-temperature performance and optimal fractional order.

Protocol Code	Optimal fractional order	Prediction model
**B**	0.186735	${\hat{x}}^{0}(k)=3987.1313e^{-2.9015(k-1)}+3987.1313$
**T**	0.009532	${\hat{x}}^{0}(k)=4992.556e^{0.14763(k-1)}+4992.556$
**TC**	0.009695	${\hat{x}}^{0}(k)=2799.168e^{-0.39926(k-1)}+2799.168$
**TA**	0.980962	${\hat{x}}^{0}(k)=41155.7421e^{-0.10661(k-1)}+41155.7421$

## Comprehensive performance evaluation system based on weighted averages

This study aims to systematically investigate the feasibility of constructing a multi-index comprehensive evaluation system. By introducing a weighted average calculation method combined with predictive outcomes, we seek to establish a comprehensive evaluation system for long-term moisture damage in asphalt mixtures, with the objective of providing quantitative decision-making support for engineering practice.

We used Zhejiang Province, China, as a case study. The annual average temperature in Zhejiang Province is 15–18 °C, the maximum temperature is 34.9–44.1 °C, and the minimum temperature is −3.6 °C. Precipitation is abundant, with an average annual rainfall of 1000–1900 mm. Zhejiang Province has a well-developed economy and highway transportation network. Therefore, the weights were 20%, 35%, 35%, and 10% for the resistance to water damage, high-temperature performance, fatigue life, and low-temperature performance, respectively. The optimal performance corresponded to 100 points, and the performance of the remaining schemes was expressed in percentages. For example, the TSR of TA was the highest (98.48%) after one freeze-thaw cycle, followed by the TC (95.67%). Thus, the TA had a score of 100 points regarding its resistance to water damage, and the TC had a score of 97.15 points. One freeze-thaw cycle corresponded to the early stage of water damage, 7 cycles was the middle stage, and 15 cycles was the late stage. The scores of the asphalt mixtures for different schemes are listed in Table 13.

**Table 13:** Scores of asphalt mixtures for different test schemes.

Protocol Code	Early stage	Medium term	Later stage
**B**	96.81	94.03	86.83
**T**	82.50	77.45	74.31
**TC**	88.82	91.28	91.87
**TA**	95.52	94.84	96.01

The findings demonstrate that when the highway is located in Zhejiang Province, China, the asphalt mixture incorporating an anti-stripping agent achieves the highest comprehensive score throughout the entire water damage evolution process, validating the remarkable superiority of the anti-stripping agent in long-term water damage resistance. The mixture without any additives exhibits significantly lower comprehensive scores at all stages compared to other formulations, indicating a pronounced deficiency in its water damage resistance. Consequently, the direct application of tuff in the surface layer of asphalt pavements is not recommended. The basalt-based asphalt mixture attains a comprehensive score of 96.81 in the initial freeze-thaw cycles, which subsequently declines to 86.83 in later stages. In contrast, the tuff mixture modified with cement maintains a stable score of 90 ± 2 throughout the entire process, confirming its performance stability under prolonged water damage conditions.

The comprehensive evaluation system proposed in this study exhibits the advantages of multi-dimensional integration, dynamic weight adjustment, and quantitative assessment criteria. Specifically, it transcends the limitations of evaluating individual indicators of asphalt mixtures in isolation, instead incorporating core performance metrics (including moisture susceptibility, high-temperature rutting resistance, intermediate-temperature fatigue resistance, and low-temperature cracking resistance) into a unified assessment framework. The system introduces regionally adaptive weighting coefficients, dynamically adjusting the weightings of each performance criterion to accommodate the requirements of different climatic zones, thereby ensuring that the final results align more closely with local conditions. Furthermore, a benchmark reference system is established, wherein the optimal performance parameters at each stage serve as the baseline (100 points). Through a relative scoring method (X = measured value/ benchmark value × 100), comparable analysis between different solutions is facilitated. Additionally, the system enables long-term, continuous monitoring of road service conditions and distress development, allowing for iterative refinement of weighting proportions to enhance the adaptability and accuracy of the evaluation framework over time.

## Conclusion

This study investigated and predicted the impact of long-term water damage on the various properties of tuff-asphalt mixtures. A multi-index evaluation system for long-term water damage of asphalt mixtures was established using the weighted average method. The following conclusions were drawn:

(1) Both additives improved the properties of tuff-asphalt mixtures to varying degrees after water damage. The anti-stripping agent enhances the bond strength at the asphalt-aggregate interface by modifying the surface characteristics of tuff aggregates, thereby demonstrating superior resistance to stripping under short-term moisture damage conditions. In contrast, cement generates products such as CaCO₃ through continuous hydration reactions, which not only inhibit moisture infiltration but also provide additional strength support, thus maintaining stable resistance to moisture-induced damage throughout the entire service life.
(2) Water damage effects lead to an increase in the viscous components of asphalt mixtures and a reduction in their elastic recovery capacity. It provides a theoretical basis for rut formation. Furthermore, the lower density characteristics of tuff aggregates constitute a critical factor contributing to the diminished fatigue life of tuff-based asphalt mixtures compared to basalt mixtures.
(3) The FGM was employed to forecast the road performance of asphalt mixtures subjected to long-term water damage. The relative errors between the predicted results and the actual experimental data were consistently maintained within 3%, demonstrating the technical feasibility of this approach for assessing the long-term water damage effects and the evolution of road performance in asphalt mixtures.
(4) The established comprehensive evaluation system for asphalt mixtures exhibits the advantages of multi-dimensional integration, dynamic weight adjustment, and quantifiable assessment criteria. This system demonstrates significant application value in both theoretical construction and engineering practice. In future research, we plan to monitor the long-term usage of the test sections, further optimise the weighting factors, and incorporate cost analysis to ensure the evaluation system is suitable for practical engineering applications.

## Supporting information

S1 Data (DOCX)
