# Supplementary material for: A multi-index evaluation system for tuff-asphalt mixtures exposed to long-term water damage based on the fractional grey prediction model
Source: PLoS One. 2025 Jul 10;20(7):e0327853. doi: 10.1371/journal.pone.0327853 (PMC12244547; doi:10.1371/journal.pone.0327853)
Supplement: S1 Data — (DOCX) [file pone.0327853.s001.docx]

Asphalt mixture test protocol.

| **Protocol Code** | **Coarse aggregate** | **Fine aggregate** | **Filler** | **Asphalt** |
| --- | --- | --- | --- | --- |
| **B** | Basalt | Limestone | Limestone powder | SBS modified asphalt |
| **T** | Tuff | Limestone | Limestone powder | SBS modified asphalt |
| **TC** | Tuff | Limestone | Limestone powder  (20% cement) | SBS modified asphalt |
| **TA** | Tuff | Limestone | Limestone powder | SBS modified asphalt  (0.6% Anti stripping agent) |

TSR of asphalt mixtures after freeze-thaw cycles. (%)

| Freeze-thaw damage cycles | TA | B | T | TC |
| --- | --- | --- | --- | --- |
| 1 | 98.48 | 89.75 | 88.73 | 95.67 |
| 3 | 96.85 | 80.04 | 77.23 | 88.21 |
| 5 | 77.34 | 71.32 | 65.02 | 85.8 |
| 7 | 73.36 | 66.76 | 59.19 | 81.83 |

Anti-rutting factors of the asphalt mixture after freeze-thaw cycles. (1Hz) (MPa)

| Freeze-thaw damage cycles | TC | T | B | TA |
| --- | --- | --- | --- | --- |
| 0 | 317 | 339 | 399 | 436 |
| 1 | 271 | 228 | 332 | 342 |
| 3 | 208 | 191 | 266 | 277 |
| 5 | 166 | 157 | 199 | 205 |
| 7 | 125 | 118 | 130 | 132 |

Anti-rutting factors of the asphalt mixture after freeze-thaw cycles. (5Hz) (MPa)

| Freeze-thaw damage cycles | TC | T | B | TA |
| --- | --- | --- | --- | --- |
| 0 | 506 | 521 | 590 | 620 |
| 1 | 473 | 430 | 501 | 518 |
| 3 | 362 | 352 | 421 | 435 |
| 5 | 279 | 261 | 341 | 371 |
| 7 | 226 | 210 | 261 | 267 |

Anti-rutting factors of the asphalt mixture after freeze-thaw cycles. (10Hz) (MPa)

| Freeze-thaw damage cycles | TC | T | B | TA |
| --- | --- | --- | --- | --- |
| 0 | 607 | 620 | 720 | 733 |
| 1 | 572 | 568 | 614 | 634 |
| 3 | 457 | 449 | 513 | 518 |
| 5 | 361 | 354 | 379 | 391 |
| 7 | 290 | 280 | 323 | 344 |

Anti-rutting factors of the asphalt mixture after freeze-thaw cycles. (20Hz) (MPa)

| Freeze-thaw damage cycles | TC | T | B | TA |
| --- | --- | --- | --- | --- |
| 0 | 777 | 774 | 864 | 875 |
| 1 | 667 | 652 | 761 | 783 |
| 3 | 573 | 556 | 639 | 671 |
| 5 | 457 | 413 | 521 | 534 |
| 7 | 374 | 356 | 411 | 430 |

Anti-rutting factors of the asphalt mixture after freeze-thaw cycles. (25Hz) (MPa)

| Freeze-thaw damage cycles | TC | T | B | TA |
| --- | --- | --- | --- | --- |
| 0 | 1064 | 1068 | 1113 | 1172 |
| 1 | 865 | 855 | 912 | 947 |
| 3 | 698 | 667 | 754 | 768 |
| 5 | 555 | 544 | 583 | 614 |
| 7 | 510 | 484 | 531 | 567 |

Phase angle of the asphalt mixture after the freeze-thaw cycles at 25 Hz. (**°**)

| Test Scheme | Phase angle after different freeze-thaw cycles $\varphi$(°) | | | | |
| --- | --- | --- | --- | --- | --- |
|  | 0 | 1 | 3 | 5 | 7 |
| B | 28.36 | 32.44 | 32.68 | 34.78 | 35.37 |
| T | 29.48 | 31.32 | 33.3 | 35.69 | 36.23 |
| TC | 29.7 | 32.62 | 34.09 | 34.35 | 35.54 |
| TA | 27.82 | 32.5 | 32.9 | 33.67 | 34.78 |

Fatigue life of asphalt mixtures after freeze-thaw cycles. (cycles)

| Freeze-thaw damage cycles | T | TC | TA | B |
| --- | --- | --- | --- | --- |
| 0 | 4417 | 5349 | 5536 | 6349 |
| 1 | 4200 | 5146 | 5103 | 6137 |
| 3 | 3991 | 4730 | 4661 | 5675 |
| 5 | 3639 | 4511 | 4510 | 5162 |
| 7 | 3455 | 4189 | 4324 | 4737 |

Low-temperature fracture energy of asphalt mixtures after freeze-thaw cycles. (J/m^2^)

| Freeze-thaw damage cycles | T | TC | TA | B |
| --- | --- | --- | --- | --- |
| 0 | 3706 | 3566 | 4042 | 3649 |
| 1 | 3466 | 3279 | 3830 | 3369 |
| 3 | 3204 | 3058 | 3470 | 2946 |
| 5 | 2934 | 3019 | 3211 | 2764 |
| 7 | 2604 | 2881 | 2833 | 2640 |

Predicted and experimental results. (Water stability) (%)

| Freeze-thaw damage cycles | B-Predictive | TA-Predictive | TC-Predictive | T-Predictive | T-Measured | B-Measured | TA-Measured | TC-Measured |
| --- | --- | --- | --- | --- | --- | --- | --- | --- |
| 1 | 89.75 | 98.48 | 95.67 | 88.73 | 88.73 | 89.75 | 98.48 | 95.67 |
| 3 | 78.94 | 91.58 | 88.2 | 75.54 | 77.23 | 80.04 | 96.85 | 88.21 |
| 5 | 71.51 | 79.86 | 85.63 | 65.42 | 65.02 | 71.32 | 77.34 | 85.8 |
| 7 | 66.76 | 73.36 | 81.83 | 59.19 | 59.19 | 66.76 | 73.36 | 81.83 |
| 9 | 63.45 | 69 | 77.49 | 55.01 | / | / | / | / |
| 11 | 60.95 | 65.77 | 72.98 | 51.95 | / | / | / | / |
| 13 | 58.96 | 63.22 | 68.49 | 49.56 | / | / | / | / |
| 15 | 57.32 | 61.13 | 64.13 | 47.62 | / | / | / | / |

Predicted and experimental results. (High temperature performance(20Hz)) (MPa)

| Freeze-thaw damage cycles | B-Predictive | TA-Predictive | TC-Predictive | T-Predictive | T-Measured | B-Measured | TC-Measured | TA-Measured |
| --- | --- | --- | --- | --- | --- | --- | --- | --- |
| 1 | 864 | 875 | 777 | 774 | 774 | 864 | 777 | 875 |
| 3 | 761 | 784 | 668 | 650 | 652 | 761 | 667 | 783 |
| 5 | 630 | 654 | 559 | 529 | 556 | 639 | 573 | 671 |
| 7 | 514 | 534 | 459 | 430 | 413 | 521 | 457 | 534 |
| 9 | 418 | 436 | 377 | 356 | 356 | 411 | 374 | 430 |
| 11 | 340 | 357 | 310 | 301 | / | / | / | / |
| 13 | 278 | 296 | 258 | 261 | / | / | / | / |
| 15 | 229 | 248 | 217 | 231 | / | / | / | / |

Predicted and experimental results. (Fatigue performance) (cycles)

| Freeze-thaw damage cycles | B-Predictive | TA-Predictive | TC-Predictive | T-Predictive | T-Measured | B-Measured | TC-Measured | TA-Measured |
| --- | --- | --- | --- | --- | --- | --- | --- | --- |
| 1 | 6349 | 5536 | 5349 | 4417 | 4417 | 6349 | 5349 | 5536 |
| 3 | 6137 | 5045 | 5082 | 4200 | 4200 | 6087 | 5146 | 5103 |
| 5 | 5654 | 4702 | 4767 | 3958 | 3991 | 5675 | 4730 | 4661 |
| 7 | 5177 | 4481 | 4455 | 3692 | 3639 | 5162 | 4511 | 4510 |
| 9 | 4727 | 4324 | 4189 | 3426 | 3455 | 4737 | 4189 | 4324 |
| 11 | 4310 | 4203 | 3973 | 3171 | / | / | / | / |
| 13 | 3926 | 4106 | 3797 | 2929 | / | / | / | / |
| 15 | 3573 | 4025 | 3652 | 2703 | / | / | / | / |

Predicted and experimental results. (Low temperature performance) (J/m^2^)

| Freeze-thaw damage cycles | B-Predictive | TA-Predictive | TC-Predictive | T-Predictive | T-Measured | B-Measured | TC-Measured | TA-Measured |
| --- | --- | --- | --- | --- | --- | --- | --- | --- |
| 1 | 3649 | 4042 | 3566 | 3706 | 3706 | 3649 | 3566 | 4042 |
| 3 | 3287 | 3830 | 3279 | 3466 | 3466 | 3369 | 3279 | 3830 |
| 5 | 2968 | 3484 | 3095 | 3213 | 3204 | 2946 | 3058 | 3470 |
| 7 | 2774 | 3159 | 2973 | 2930 | 2934 | 2764 | 3019 | 3211 |
| 9 | 2640 | 2861 | 2891 | 2607 | 2604 | 2640 | 2881 | 2833 |
| 11 | 2539 | 2588 | 2835 | 2236 | / | / | / | / |
| 13 | 2458 | 2340 | 2798 | 1809 | / | / | / | / |
| 15 | 2392 | 2115 | 2772 | 1316 | / | / | / | / |
